# Sex and body mass index dependent associations between serum 25-hydroxyvitamin D and pulse pressure in middle-aged and older US adults

**DOI:** 10.1038/s41598-021-88855-8

**Published:** 2021-05-11

**Authors:** Jung Hyun Kwak, Yoon-Hyeong Choi

**Affiliations:** 1 Department of Preventive Medicine, Gachon University College of Medicine, 155 Gaetbeol-ro, Yeonsu-gu, Incheon, 21999 Republic of Korea; 2 Department of Food and Nutrition, Eulji University, 553 Sanseong-daero, Sujeong-gu, Seongnam, 13135 Republic of Korea; 3 Gachon Advanced Institute for Health Sciences and Technology, Incheon, Republic of Korea

**Keywords:** Environmental impact, Statistical methods, Risk factors, Hypertension, Epidemiology

## Abstract

High pulse pressure (PP) is a valid indicator of arterial stiffness. Many studies have reported that vitamin D concentration is inversely associated with vascular stiffening. This association may differ depending on sex and body mass index (BMI). This study investigated the associations between vitamin D and PP and evaluated whether these associations differ according to sex and BMI, using data for individuals aged ≥ 50 years from the National Health and Nutrition Examination Survey (NHANES) 2007–2010. Serum 25-hydroxyvitamin D (25(OH)D) concentrations were used as biomarkers of vitamin D levels. High PP was defined as ≥ 60 mmHg. Total 25(OH)D concentrations were dose-dependently associated with lower odds ratios (ORs) for high PP (*p-*trend = 0.01), after controlling for sociodemographic, behavioral, and dietary factors. When stratified by sex, there was a dose-dependent association between total 25(OH)D concentrations and lower risk of high PP (*p-*trend < 0.001) in females, but not in males. When stratified by BMI, there was a dose-dependent association between total 25(OH)D concentrations and lower risk of high PP (*p-*trend < 0.001) in non-overweight subjects, but not in overweight subjects. Improving the vitamin D status could delay elevation of PP and vascular stiffening in female and non-overweight subjects.

## Introduction

Hypertension is an important public health issue that is highly prevalent in the elderly population. According to the 2015–2016 National Center for Health Statistics (NCHS) data, the prevalence of hypertension was 29.0% among adults, and it increased with age (63.1% in adults aged 60 years and older)^1^. In the elderly population, hypertension is characterized by an elevated systolic blood pressure (SBP) with a normal or low diastolic blood pressure (DBP) caused by age-associated stiffening of the large arteries. Thus, pulse pressure (PP)^2^, defined as the difference between SBP and DBP, is a valid indicator of arterial stiffness in older adults^3^. Because increased arterial stiffness is known to be associated with an increased risk of cardiovascular disease as well as overall mortality^4,5^, PP management is very important in the elderly population to prevent cardiovascular diseases such as stroke, heart attack, and heart failure^6,7^.

Several studies have reported that vitamin D improves endothelial function^8^, and vitamin D levels are inversely associated with vascular stiffening^9,10^. It is possible that vitamin D improves general arterial health and increases arterial elasticity, resulting in improved arterial compliance and decreased PP^11^. Although there is abundant plausible biological evidence to support that vitamin D plays a role in hypertension and vascular health, previous epidemiological and intervention studies have presented inconsistent results^12,13^.

Therefore, the primary goal of the present study was to investigate the association between vitamin D and PP in middle-aged and older adults using data from the National Health and Nutrition Examination Survey (NHANES) 2007–2010. The secondary goal of the study was to evaluate if sex and body mass index (BMI) influence the association between vitamin D and PP.

## Results

### Descriptive statistics

A total of 4565 subjects (2316 males and 2249 females) were included in the analysis. Table 1 shows the general characteristics of the subjects according to sex. In males, the mean ± standard error (± SE) age was 62.4 (± 0.2) years, and 55.4% of this group had hypertension. The geometric mean (95% CIs) of the total 25-hydroxyvitamin D (25(OH)D), 25(OH)D_3_, and 25(OH)D_2_ concentrations in males were 64.4 nmol/L (95% CIs 62.4, 66.4), 59.7 nmol/L (95% CIs 57.9, 61.6), and 2.22 nmol/L (95% CIs 2.09, 2.36), respectively. In females, the mean (± SE) age was 63.5 (± 0.3), and 58.4% of this group had hypertension. The geometric mean (95% CIs) of total 25(OH)D, 25(OH)D_3_ and 25(OH)D_2_ concentrations in women were 66.7 nmol/L (95% CIs 64.4, 69.1), 60.1 nmol/L (95% CIs 58.0, 62.2), and 2.49 nmol/L (95% CIs 2.29, 2.71), respectively. The males were significantly younger and had significantly lower total 25(OH)D, 25(OH)D_2_, and PP values and higher DBP, height, weight, physical activity, total energy intake, potassium, calcium, magnesium, and sodium intake values than females. In addition, alcohol consumption, smoking status, and education levels were significantly different depending on sex.

**Table 1:** Participants characteristics by sex.

Characteristics	Males	Females	*p*
No. of participants	2316	2249	–
Total 25(OH)D (nmol/L)^a^	64.4 (62.4–66.4)	66.7 (64.4–69.1)	0.02
25(OH)D_3_ (nmol/L)^a^	59.7 (57.9–61.6)	60.1 (58.0–62.2)	0.69
25(OH)D_2_ (nmol/L)^a^	2.22 (2.09–2.36)	2.49 (2.29–2.71)	0.005
Systolic blood pressure (mmHg)	128 (± 0.4)	128 (± 0.5)	0.96
Diastolic blood pressure (mmHg)	72.0 (± 0.4)	68.6 (± 0.5)	< 0.001
Pulse pressure (mmHg)	56.1 (± 0.5)	59.5 (± 0.7)	< 0.001
Age (years)	62.4 (± 0.2)	63.5 (± 0.3)	< 0.001
Height (cm)	175 (± 0.2)	161 (± 0.2)	< 0.001
Weight (kg)	90.0 (± 0.7)	76.2 (± 0.6)	< 0.001
Physical activity (METs-h)	58.1 (± 2.8)	28.9 (± 1.4)	< 0.001
Total energy intake (kcal)	2280 (± 30)	1700 (± 20)	< 0.001
Dietary potassium (mg)	3070 (± 40)	2480 (± 30)	< 0.001
Dietary calcium (mg)	990 (± 20)	850 (± 10)	< 0.001
Dietary magnesium (mg)	328 (± 4.4)	267 (± 3.9)	< 0.001
Dietary sodium (mg)	3820 (± 60)	2820 (± 40)	< 0.001
**Race/ethnicity (%)**			0.39
Non-Hispanic White	79.1	79.1	
Non-Hispanic Black	8.1	9.0	
Mexican American	5.1	4.9	
Other ethnicity	7.7	7.0	
**Alcohol consumption (%)**			< 0.001
Never	29.1	41.0	
< once a week	27.1	34.2	
1–2 days/week	17.1	10.8	
3–4 days/week	10.0	5.5	
≥ 5 days/week	16.6	8.5	
**Smoking status (%)**			< 0.001
Never	40.1	57.3	
Former	43.4	30.0	
Current	16.5	12.7	
**Season of examination (%)**			0.68
November–April	34.9	34.5	
May–October	65.1	65.5	
**Education (%)**			< 0.001
< 9th grade	9.0	7.1	
≤ High school graduate	35.3	39.9	
> High school graduate	55.7	53.0	
Hypertension (%)	55.4	58.4	0.12
Diabetes (%)	16.8	15.4	0.36

Survey t-test for continuous variables and survey (Rao-Scott) χ^2^ test for categorical variables were used.

^a^Geometric mean (95% confidence intervals) are presented.

### Vitamin D and blood pressure in all subjects

Table 2 presents the results of multiple linear regression analyses for the associations of vitamin D with blood pressure. In all sequential models, higher total 25(OH)D and 25(OH)D_3_ concentrations were dose-dependently associated with lower mean SBP and PP (*p-*trend < 0.05). In the fully adjusted model (model B), subjects with sufficient total 25(OH)D (≥ 75 nmol/L) had a significant 2.95 (95% CIs 1.39, 4.50) mmHg lower SBP and 2.52 (95% CIs 1.10, 3.95) mmHg lower PP than did those with deficient total 25(OH)D (< 50 nmol/L). The results for the associations between 25(OH)D_3_ concentrations and blood pressure were similar to the associations between total 25(OH)D concentrations and blood pressure.

**Table 2:** Adjusted multiple linear regression and 95% CIs of blood pressure by vitamin D concentrations in all subjects aged ≥ 50 years.

Variables	Model A			Model B		
	SBP	DBP	PP	SBP	DBP	PP
**All subjects (n = 4565)**						
25(OH)D_3_ (nmol/L)						
< 50	0 (Reference)	0 (Reference)	0 (Reference)	0 (Reference)	0 (Reference)	0 (Reference)
50–74.9	− 1.16 (− 2.67, 0.36)	0.94 (0.05, 1.82)*	− 2.09 (− 3.69, − 0.50)*	− 0.60 (− 2.04, 0.85)	0.65 (− 0.27, 1.58)	− 1.25 (− 2.84, 0.33)
≥ 75	− 3.36 (− 5.05, − 1.66)***	0.43 (− 0.61, 1.46)	− 3.78 (− 5.36, − 2.20)***	− 2.43 (− 4.14, − 0.72)**	− 0.08 (− 1.17, 1.01)	− 2.35 (− 4.00, − 0.70)**
*p*-trend	< 0.001	0.49	< 0.001	0.005	0.78	0.006
Total 25(OH)D (nmol/L)						
< 50	0 (Reference)	0 (Reference)	0 (Reference)	0 (Reference)	0 (Reference)	0 (Reference)
50–74.9	− 1.09 (− 2.66, 0.48)	0.09 (− 0.97, 1.15)	− 1.45 (− 2.99, 0.09)^†^	− 0.56 (− 2.10, 0.97)	0.09 (− 0.98, 1.16)	− 0.65 (− 2.14, 0.83)
≥ 75	− 3.84 (− 5.46, − 2.22)***	0.36 (− 0.63, 1.35)	− 3.93 (− 5.45, − 2.42)***	− 2.95 (− 4.50, − 1.39)**	− 0.42 (− 1.57, 0.72)	− 2.52 (− 3.95, − 1.10)**
*p*-trend	< 0.001	1.00	< 0.001	< 0.001	0.36	< 0.001

Model A was adjusted for age, sex, and race/ethnicity.

Model B: Model A + further adjusted for education, season of examination, physical activity, alcohol consumption, smoking status, dietary covariates (intakes of total energy, potassium, calcium, magnesium, and sodium), height, weight, and diabetes.

***p < 0.001, **p < 0.01, *p < 0.05, ^†^*p* < 0.1 compared with vitamin D levels < 50 nmol/L.

### Vitamin D and high PP in all subjects

Table 3 presents the results of multiple logistic regression analyses of the associations of vitamin D with high PP. In all sequential models, higher total 25(OH)D and 25(OH)D_3_ concentrations were dose-dependently associated with lower odd ratios (ORs) for high PP (*p-*trend < 0.05). In the fully adjusted models (model B), the ORs for high PP were 0.76 (95% CIs 0.61, 0.95) in the higher total 25(OH)D concentrations (≥ 75 nmol/L) group compared to the group with lower total 25(OH)D concentrations (< 50 nmol/L). The results for the associations between 25(OH)D_3_ concentrations and high PP were similar to the associations between total 25(OH)D concentrations and high PP. These associations were similar after adjusting for additional dietary factors such as the intakes of total fat, saturated fat, and cholesterol (data not shown).

**Table 3:** Adjusted multiple logistic regression and 95% CIs of high pulse pressure by vitamin D concentrations in all subjects aged ≥ 50 years.

Variables	High PP	
	Model A	Model B
**Total subject (n = 4565)**		
25(OH)D_3_ (nmol/L)		
< 50	1 (Reference)	1 (Reference)
50–74.9	0.74 (0.56, 0.94)*	0.82 (0.64, 1.05)
≥ 75	0.65 (0.53, 0.80)**	0.77 (0.61, 0.98)*
*p*-trend	< 0.001	0.03
Total 25(OH)D (nmol/L)		
< 50	1 (Reference)	1 (Reference)
50–74.9	0.83 (0.68, 1.01)^†^	0.90 (0.74, 1.11)
≥ 75	0.65 (0.53, 0.79)**	0.76 (0.61, 0.95)*
*p*-trend	< 0.001	0.01

Model A was adjusted for age, sex, and race/ethnicity.

Model B: Model A + further adjusted for education, season of examination, physical activity, alcohol consumption, smoking status, dietary covariates (intakes of total energy, potassium, calcium, magnesium, and sodium), height, weight, and diabetes.

**p < 0.001, *p < 0.05, ^†^*p* < 0.1 compared with vitamin D levels < 50 nmol/L.

### Total 25(OH)D concentration and blood pressure stratified by sex and BMI

**Figure 1:**
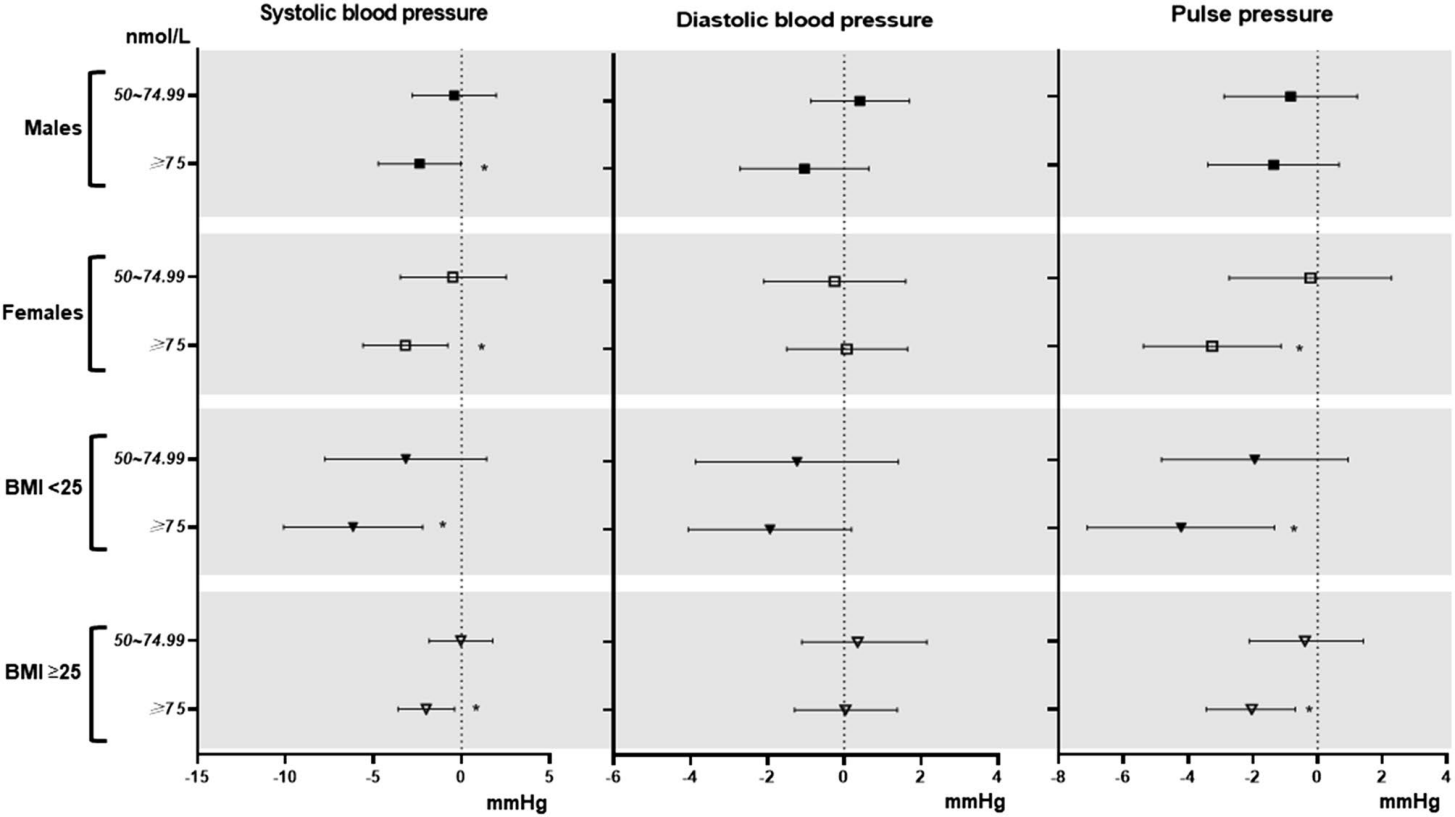
Multiple linear regression of blood pressure with vitamin D concentrations in the subgroups by sex and BMI. *p*-trend < 0.05 considered significant. Adjusted for age, sex, race/ethnicity, education, season of examination, physical activity, alcohol consumption, smoking status, dietary covariates (intakes of total energy, potassium, calcium, magnesium, and sodium), height, weight, and diabetes.

Figure 1 shows the results of multiple linear regression analyses for the association of total 25(OH)D levels with blood pressure stratified by sex and BMI in the fully adjusted models.

When stratified by sex, the SBP decreased significantly in a dose-dependent manner with an increase in the total 25(OH)D concentration in males (*p-*trend = 0.02), and the SBP and PP decreased significantly in a dose-dependent manner with an increase in the total 25(OH)D concentration in females (*p-*trend = 0.003 and 0.001). The males with sufficient total 25(OH)D concentrations had a significant 2.39 mmHg (95% CIs 0.05, 4.74) lower SBP than those with deficient total 25(OH)D concentrations, while the females with sufficient total 25(OH)D concentrations had a significant 3.19 mmHg (95% CIs 0.79, 5.59) lower SBP and 3.26 mmHg (95% CIs 1.13, 5.38) lower PP than those with deficient total 25(OH)D concentrations.

When stratified by BMI, significant dose-dependent decreases in SBP and PP (*p-*trend = 0.002 and 0.005) with total 25(OH)D concentrations were observed in non-overweight subjects, and significant dose-dependent decreases in SBP and PP (*p-*trend = 0.006 and 0.003) with total 25(OH)D concentrations were observed in overweight subjects. Non-overweight subjects with sufficient total 25(OH)D concentrations had a significant 6.16 mmHg (95% CIs 2.22, 10.1) lower SBP and 4.22 mmHg (95% CIs 1.33, 7.12) lower PP than those with deficient total 25(OH)D concentrations. Overweight subjects with sufficient total 25(OH)D concentrations had a significant 2.01mmHg (95% CIs 0.41, 3.60) lower SBP and 2.04 mmHg (95% CIs 0.64, 3.44) lower PP than those with deficient total 25(OH)D concentrations.

### Total 25(OH)D concentration and high PP stratified by sex and BMI

**Figure 2:**
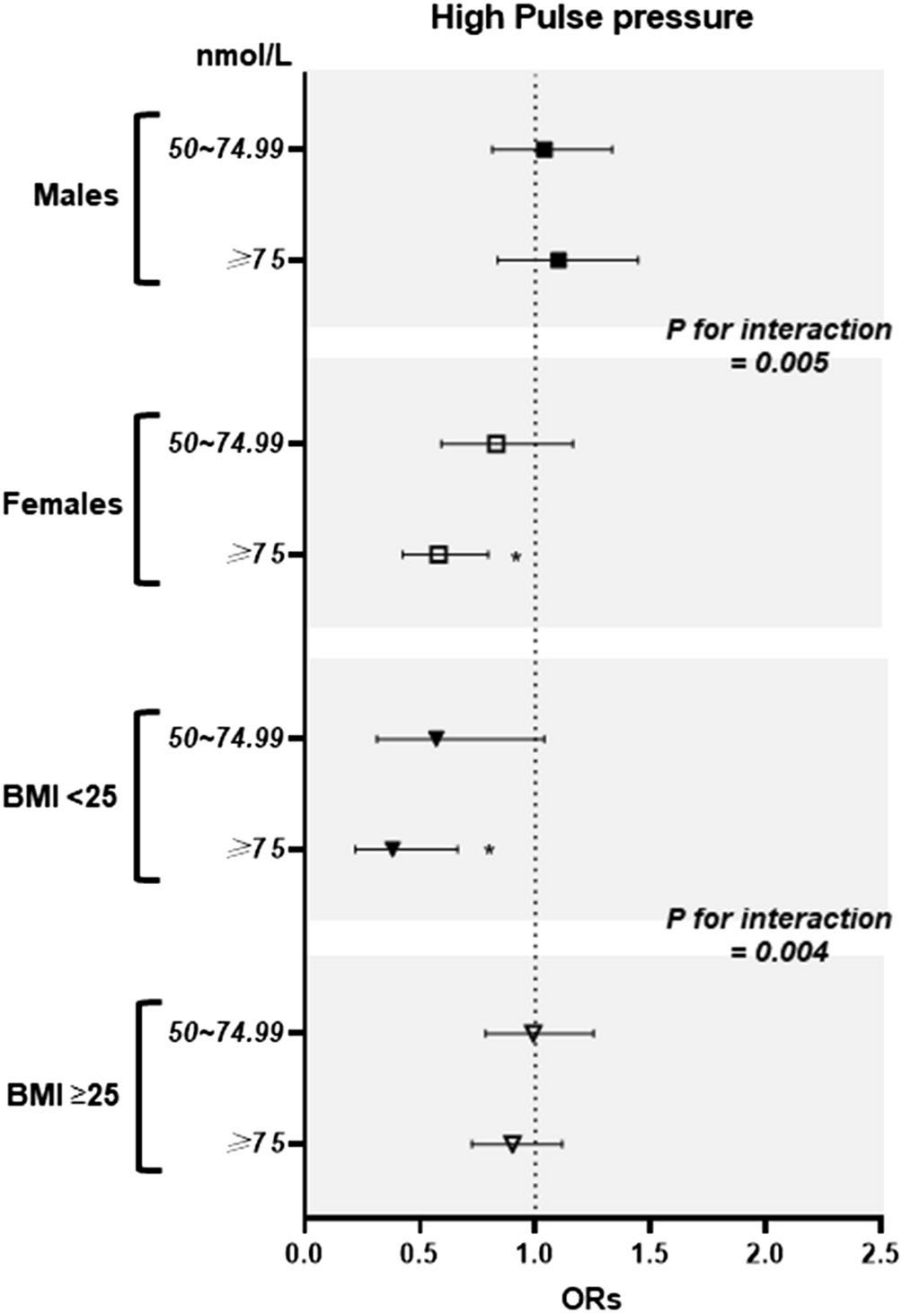
Multiple logistic regression of high pulse pressure with total 25(OH)D concentrations in the subgroups by sex and BMI. *p-trend < 0.05 considered significant. Adjusted for age, sex, race/ethnicity, education, season of examination, physical activity, alcohol consumption, smoking status, dietary covariates (intakes of total energy, potassium, calcium, magnesium, and sodium), height, weight, and diabetes.

Figure 2 shows the results of the multiple logistic regression analyses for the associations of total 25(OH)D concentration with high PP stratified by sex and BMI in the fully adjusted models. Dose-dependent associations between total 25(OH)D concentration and high PP (*p*-trend < 0.001 and < 0.001) were observed in females and in non-overweight subjects.

When stratified by sex, females had a significantly lower OR for high PP were 0.58 (95% CIs 0.42, 0.79) in subjects with sufficient total 25(OH)D concentrations (versus deficient subjects), whereas males had a higher OR for high PP were 1.10 (95% CIs 0.84, 1.45) in subjects with sufficient total 25(OH)D concentrations. The associations between total 25(OH)D concentration and high PP significantly differed according to sex (*p* for interaction = 0.005).

When stratified by BMI, non-overweight subjects had a significantly lower OR for high PP were 0.38 (95% CIs 0.22, 0.67) in subjects with sufficient total 25(OH)D concentrations (versus deficient subjects), whereas overweight subjects showed no significant difference in the risk for high PP according to the total 25(OH)D concentrations. The associations between total 25(OH)D concentration and high PP significantly differed according to BMI (*p* for interaction = 0.004).

Participant characteristics according to the three BMI classifications are presented in Supplemental Table 1. In sensitivity analysis, when stratified by the three BMI classifications (obese, overweight, and, non-overweight), subjects with overweight and obesity showed no significant difference in the risk of high PP based on total 25(OH)D concentrations in Supplemental Table 2.

## Discussion

In a nationally representative sample of middle-aged and older adults, we found that vitamin D sufficiency is associated with a lower risk of high PP. When stratified by sex and BMI, females and non-overweight subjects presented significant associations between vitamin D sufficiency and lower risk of high PP, whereas the risk for high PP did not vary significantly with the 25(OH)D concentrations in males and overweight subjects. Aging is associated with a progressive increase in vascular stiffness and PP^2^. High PP has been attributed to elastic fiber degeneration and aortic dilation, which transfers the hemodynamic load to stiffer collagen^14^; this condition is associated with an increased risk for cardiovascular disease and overall mortality^15,16^. Therefore, effective management of PP is very important to prevent cardiovascular disease and to ensure a healthy life in the elderly^6,7^. Several studies have reported that serum vitamin D concentrations are inversely associated with vascular stiffening^9,10^. It is possible that vitamin D improves general arterial health and increases arterial elasticity, which results in improvement in arterial compliance and decrease in PP^11^. Additionally, vitamin D may improve endothelial function by modulating inflammation-related cytokines and by increasing the expression of vascular endothelial growth factors^17^. One experimental study conducted by Pelham et al. investigated whether vitamin D deficiency leads to impaired function in the resistance artery by altering the phenotype of perivascular adipose tissue (PVAT). They reported that vitamin D deficiency enhances angiotensin II-induced vasoconstriction and impairs the normal ability of PVAT, whereas, the intake of supplementary vitamin D can protect against vascular dysfunction caused by impaired PVAT function due to hypoxia^18^.

Although epidemiological evidence consistently shows that vitamin D affects musculoskeletal health^19^, there has been a recent debate regarding the role of vitamin D deficiency in blood pressure based on conflicting observational and intervention studies^20–22^. Inconsistent results of the association between vitamin D and PP, particularly in clinical studies, may have been caused by differences in the study population and design, i.e., differences in the definition of vitamin D deficiency, supplementary vitamin D dose, and/or serum 25(OH)D concentrations, along with differences in the study period^23–26^. Nevertheless, a clinical study by Raed et al. reported that high doses of vitamin D (> 18,000 IU) for women with vitamin D deficiency (< 20 ng/mL) were effective in reducing PP^27^, which may, in part, support our hypothesis.

The current study suggests that the role of vitamin D deficiency in PP may differ depending on sex and BMI. Accordingly, the following interpretations could be made. The differences in results between males and females observed in the current study may be due to the fact that males have a higher visceral fat percentage than females. An increase in the visceral fat component of the body leads to an increase in the secretion of free fatty acids^28^, which degrades vascular function^29^. Males also have a higher level of insulin resistance than females^28^. Increased insulin resistance is a major risk factor that lowers vascular elasticity^30^. In addition, the androgen levels in males are higher than that in females. A study reported that androgens may upregulate the renin-angiotensin system and may play an important role in the pathogenesis of hypertension, which could cause endothelial injury^31^. Moreover, a recent study conducted by Varakantham et al. suggested the sex-specific role of CYP24A1 genetic variants related to vitamin D metabolism and reported that the risk of hypertension is increased in men with the rs2762939 CC variant; however, this risk of hypertension is decreased in women with the rs2762939 CC variant. This difference is thought to be due to the different effects of CYP24A1 genetic variants on 25(OH)D and renin concentrations in a sex-dependent manner^32^. Overall, males are metabolically vulnerable to lower vascular elasticity compared to females; thus, the vascular elasticity status may not reflect the vitamin D activity.

Different findings according to weight may be explained by the fact that vitamin D is a fat-soluble vitamin, and is deposited in fat cells; this leads to a decrease in the bioavailability of vitamin D in obese individuals^33^. Obesity may reduce the elasticity of blood vessels by increasing oxidative stress and inflammation^34^. A recent study suggested that the effect variations of vitamin D are due to the differences in volume of distribution^35^. Obese individuals with a high volume of distribution require a higher dose of vitamin D compared to non-obese individuals^36^. Differences in the volume of distribution and in the bioavailability of vitamin D depending on body weight, and decrease in vascular elasticity due to obesity status, may possibly explain why an association between vitamin D concentrations and blood pressure parameters was not observed in overweight subjects in our study.

Several major strengths of this study should be noted. The primary strength was the use of a nationally representative survey of middle-aged and older adults in the US, and the inclusion of a large sample size. Vitamin D concentration was assessed using serum 25(OH)D and 25(OH)D_3_ concentrations, which are accepted as biomarkers of the vitamin D status and vitamin D intake over a period of 4 years^37^^,^^38^; the concentrations were measured using UHPLC-MS/MS, which is an accurate and precise method. In addition, our models included the adjustment for various potential confounders, including sociodemographic, behavioral, and dietary factors, and also included interaction terms to reflect participants’ heterogeneity.

Several limitations of the results must also be considered. First, because of the cross-sectional design, we cannot infer temporal causality. Second, findings from the large proportion of non-Hispanic whites in the study population should be taken with caution when generalizing to other ethnic populations. Third, although we adjusted our model for a variety of covariates, there is a possibility of residual confounding. For example, parathyroid hormone is thought to be a factor that can affect the association between serum vitamin D concentration and blood pressure^39^, although we did not adjust for serum parathyroid hormone due to the lack of availability of pertinent data in the NHANES 2007–2010. Additionally, nitric oxide may be generated in the skin through ultraviolet (UV)-A radiation exposure^40^ and regulated by physical exercise^41,42^, which may affect the reduction in vascular stiffness. However, we did not adjust for subcutaneous nitrogen compounds due to no available data in the NHANES. Fourth, it might be argued that our observed associations may differ depending on race/ethnicities and/or season of examination. Indeed, in our sensitivity analyses, the associations between total 25(OH)D and high PP were stronger in non-Hispanic Whites (see Supplemental Table 3) and during the warm season (see Supplemental Table 4). As solar UV radiation doses are high during the summer and there is an effect of solar UV radiation on blood pressure due to the release of nitric oxide, one needs to consider different seasons while interpreting our findings. Finally, although the NHANES includes dietary data for the first and the second day, we used dietary data for the first day alone because the method used to obtain dietary data on the second day (phone interview) differed from that used on the first day (face-to-face interview).

In summary, this study shows that vitamin D sufficiency is associated with a lower risk of high PP, possibly due to decreased arterial stiffness, in middle-aged and older adults. In particular, the association between vitamin D status and a lower risk of high PP was stronger among females and non-overweight subjects. Adequate sunlight exposure (5–30 min, 2–3 time/week)^43^, intake of foods naturally high in vitamin D (sun-dried mushrooms; egg yolks; oily fish such as salmon, sardines, mackerel; and meat)^44^ and foods fortified with vitamin D (milk and cereals)^45^, and vitamin D supplementation are likely to improve the vitamin D status and delay the elevation of PP and vascular stiffening in middle-aged and older adults.

## Methods

### Study population

The data used in this study pertained to participants aged ≥ 50 years and were obtained from the NHANES 2007–2010. The NHANES, conducted by the Centers for Disease Control and Prevention (CDC), is a health-related survey research program that assesses the health and nutritional status of a sample of the national civilian non-institutionalized United States (US) population. The survey uses a complex, multi-stage, stratified, clustered design with oversampling of Hispanic, non-Hispanic Black, and low-income White populations, as well as those aged ≥ 80 years. The survey includes interviews based on demographic, socioeconomic, and dietary questionnaires, as well as medical interviews and physical examinations including medical, dental, and physiological measurements^46,47^. The current study used the two most recent NHANES cycles (2007–2010), which included data on serum vitamin D concentration. We did not include prior NHANES cycles because their vitamin D concentration data were obtained using a method (i.e., radio-immunoassay) that was different from the one used in the 2007–2010 NHANES cycles.

**Figure 3:**
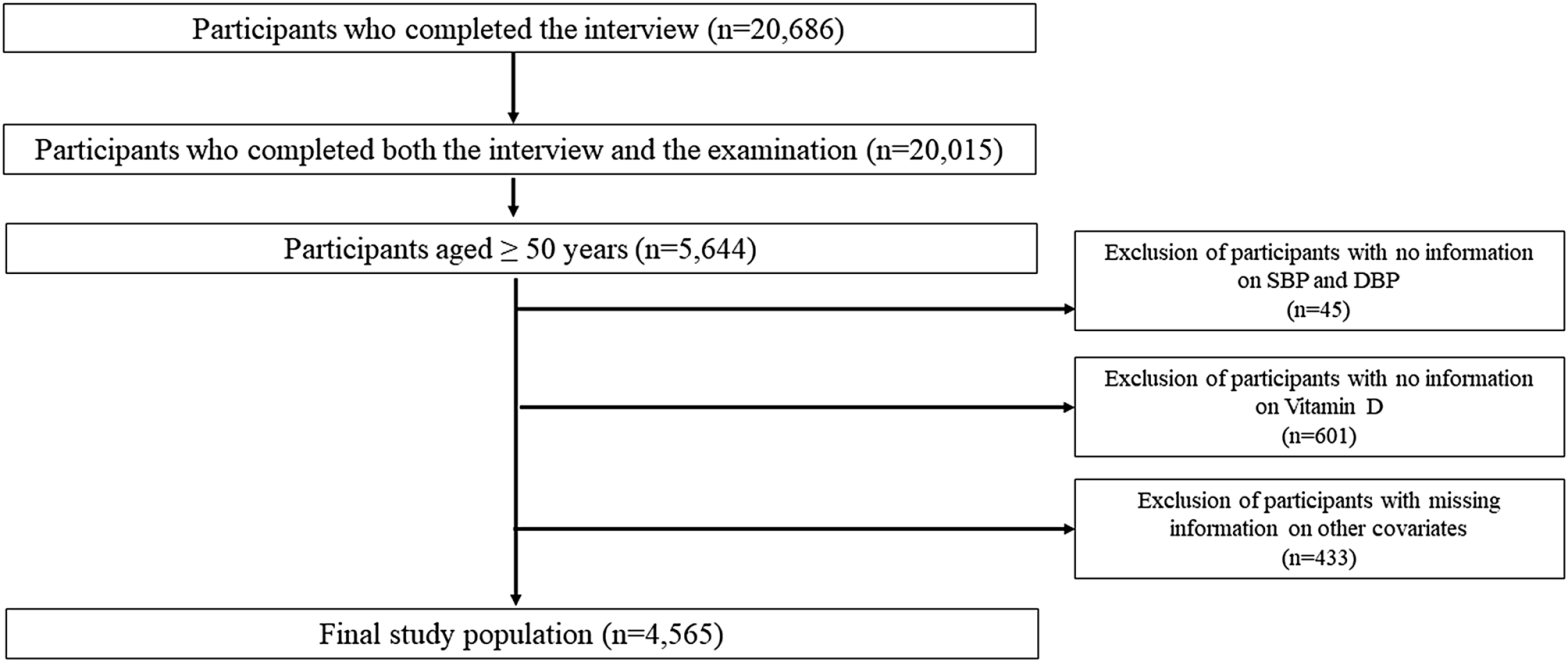
Description of the study population.

Among the initially enrolled subjects (n=20686) in the NHANES 2007–2010, complete data on both interviews and examinations were available for 20015 participants. For the present study, we selected adults aged ≥ 50 years, which resulted in 5644 participants. We additionally excluded subjects lacking information on SBP, DBP (n=45), serum vitamin D concentration (n=601), and other covariates (n=433); a total of 4565 subjects (2316 males and 2249 females) were included in our analysis. Details are illustrated in Figure 3. The study protocol was approved by the National Centers for Health Statistics Research Ethics Review board (Continuation of Protocol #2005-06). Written informed consent was obtained from all the NHANES participants. The NHANES is a publicly available dataset. Additional details on study procedures, data documentation, and questionnaires are available elsewhere^46,47^.

### Measurement of 25-hydroxyvitamin D

Blood samples for the measurement of serum vitamin D concentration were collected from participants at mobile examination centers (MECs). The blood samples were immediately frozen at − 30 °C and were subsequently shipped to the National Center for Environmental Health (CDC, Atlanta, Georgia, USA) for analysis.

The serum (25(OH)D_3_ and 25(OH)D_2_ concentrations were measured using ultra-high performance liquid chromatography-tandem mass spectrometry (UHPLC-MS/MS; ThermoElectron Corp, West Palm Beach, FL)^37^^,^^38^. Total 25(OH)D concentrations (in SI units of nmol/L) were calculated by adding the 25(OH)D_3_ and the 25(OH)D_2_ concentrations. If information on either the 25(OH)D_3_ or 25(OH)D_2_ concentrations was not available, then the total 25(OH)D value was not computed. The coefficient of variation for the NHANES 2007–2010 was in the range of 3.0–3.8% for 25(OH)D_3_, and 3.8–6.5% for 25(OH)D_2_ estimations. The range of serum concentrations was 5.41–213 nmol/L for total 25(OH)D, 3.02–212 nmol/L for 25(OH)D_3,_ and 1.45–183 nmol/L for 25(OH)D_2_. For statistical analysis, participants were categorized into three groups based on the total 25(OH)D and 25(OH)D_3_ concentrations, as follows: deficient, < 50 nmol/L; suboptimal, 50–74.9 nmol/L; and sufficient, ≥ 75 nmol/L, based on the cut-off points recommended by the US Endocrine Society^48^. We did not analyze 25(OH)D_2_ concentrations separately because the detection range for the analyte was very low.

### Blood pressure

SBP and DBP were measured at the MECs using the Omron HEM 907 XL apparatus (Omron Healthcare, Kyoto, Japan). Blood pressure was measured after the participant had rested for 5 min in a seated position; the maximum inflation level was recorded. We computed the mean SBP and DBP of three consecutive SBP and DBP values. PP was calculated as the difference between SBP and DBP, and high PP was defined as a PP value of ≥ 60 mmHg^49^.

### Body mass index

BMI was computed as weight in kilograms divided by height in meters squared (kg/m^2^). We defined “overweight” as a BMI of ≥ 25 kg/m^2^ and “non-overweight” as a BMI of < 25 kg/m^2^ in main analysis.

### Covariates

We considered participants’ age, height, weight, race/ethnicity, education, season of examination, physical activity, alcohol consumption, smoking status, diabetes, and dietary intake (total energy, potassium, calcium, magnesium, and sodium) as potential confounders. Age was categorized as 20–29, 30–39, 40–49, 50–59, 60–69, and ≥ 70 years. Race/ethnicity was categorized as non-Hispanic White (reference), Mexican American, non-Hispanic Black, and others. Education was classified as less than 9th grade (reference), high school graduate or less, and more than a high school graduate. Smoking status was classified as never (reference), former, or current smoker. Alcohol consumption was categorized as never (reference), < 1 day a week, 1–2 days/week, 3–4 days/week, or ≥ 5 days/week. The season of examination was classified as November–April (reference) or May–October. Diabetes was defined as a self-reported physician diagnosis or the current use of antidiabetic medication. Physical activity was self-reported for three activity types (walking, moderate activity, and vigorous activity), and weekly metabolic equivalent (MET) values for each activity type were calculated as the MET value × day × min. Total MET scores were computed by summing the weekly MET values for each activity type^50^. Information on dietary intake (total energy, potassium, calcium, magnesium, and sodium) was obtained through a 24-h dietary recall interview. The 24-h recall data were analyzed using USDA's Food and Nutrient Database for Dietary Studies as a food composition database^51^.

### Statistical analysis

Data analyses were performed using the SAS survey procedure (version 9.4; SAS Institute Inc., Cary, NC) to account for the complex survey design and sample weights of the NHANES 2007–2010. All *p*-values were two-sided, and a *p-*value of < 0.05 was considered statistically significant.

In the descriptive analysis, we presented weighted mean and SE for continuous variables and weighted percentages (%) for categorical variables. We further used the survey t-test for evaluating continuous variables and the Rao-Scott χ^2^ test for evaluating categorical variables. Serum 25(OH)D, 25(OH)D_2_, and 25(OH)D_3_ concentrations were right-skewed, and thus presented as geometric mean.

Pearson correlations between 25(OH)D (total 25(OH)D and 25(OH)D_3_) concentration and blood pressure were examined (r = − 0.03, p = 0.08 for total 25(OH)D; r = − 0.04, p = 0.005 for 25(OH)D_3_). The associations between 25(OH)D (total 25(OH)D and 25(OH)D_3_) concentration and blood pressure were examined using multiple linear regression models (PROC SURVEYREG). We evaluated two sequential models: model A adjusted for age, sex, and race/ethnicity; and model B adjusted for model A covariates plus season of examination, physical activity, alcohol consumption, smoking status, dietary covariates (intakes of total energy, potassium, calcium, magnesium, and sodium), height, weight, and diabetes. In addition, the associations between 25(OH)D concentration and high PP were examined using multiple logistic regression models (PROC SURVEYLOGISTIC).

All regression and logistic analyses were evaluated in subgroups stratified by sex (males vs. females) or BMI (≥ 25 vs. < 25 kg/m^2^). In addition, we estimated multiplicative interactions between serum 25(OH)D concentration and sex or BMI and computed the interaction *p* value using the Wald test.

### Sensitivity analyses

First, when we modeled subgroups stratified by BMI, we used an alternative classification of three levels of BMI (obese; BMI ≥ 30 kg/m^2^, overweight; 25–29.99 kg/m^2^, and non-overweight; BMI < 25 kg/m^2^). Second, we performed sensitivity analysis on the association between total vitamin D levels and PP in subgroups stratified by race/ethnicity (non-Hispanic White, non-Hispanic Black, Mexican American, and other ethnicity) and the season of examination (November–April and May–October). Third, we conducted another sensitivity analysis after adjusting for additional dietary factors such as total fat, saturated fat, and cholesterol as potential confounders because these dietary factors are linked to blood pressure and are associated with elevated cardiovascular disease^52–54^.

## Supplementary Information

Supplementary Tables.

## Data Availability

The datasets generated during and/or analyzed during the current study are available in the NHANES repository [https://wwwn.cdc.gov/nchs/nhanes/Default.aspx].
