# Supplementary material for: Sex and body mass index dependent associations between serum 25-hydroxyvitamin D and pulse pressure in middle-aged and older US adults
Source: Sci Rep. 2021 May 11;11:9989. doi: 10.1038/s41598-021-88855-8 (PMC8113426; doi:10.1038/s41598-021-88855-8)
Supplement: Supplementary file 1 — Supplementary Tables. [file 41598_2021_88855_MOESM1_ESM.docx]

Supplemental table 1. Participants characteristics by the three BMI classifications

| Characteristics | Normal weight  (< 25 kg/m^2^)  ( | Overweight  (25~29.99 kg/m^2^) | Obese  (≥ 30 kg/m^2^)  Obesity  (≥ 30 kg/m^2^) | *P* |
| --- | --- | --- | --- | --- |
| No. of participants | 1053 | 1679 | 1833 | - |
| Total 25(OH)D (nmol/L)^§^ | 71.6 (68.4-74.7) | 68.1 (66.2-70.1) | 59.7 (57.7-61.8) | <.001 |
| 25(OH)D_3_ (nmol/L)^§^ | 65.6 (62.8-68.3) | 62.7 (60.6-64.8) | 53.9 (52.2-55.7) | <.001 |
| 25(OH)D_2_ (nmol/L)^§^ | 2.51 (2.31-2.71) | 2.33 (2.17-2.49) | 2.29 (2.11-2.47) | 0.02 |
| Systolic blood pressure (mmHg) | 128 (±0.7) | 128 (±0.6) | 128 (±0.5) | 0.33 |
| Diastolic blood pressure (mmHg) | 69.8 (±0.5) | 70.6 (±0.4) | 70.1 (±0.6) | 0.77 |
| Pulse pressure (mmHg) | 57.9 (±0.8) | 57.5 (±0.7) | 58.2 (±0.6) | 0.54 |
| Age (years) | 63.8 (±0.4) | 63.2 (±0.3) | 62.3 (±0.3) | <.001 |
| Height (cm) | 167 (±0.3) | 169 (±0.3) | 168 (±0.3) | 0.09 |
| Weight (kg) | 62.4 (±0.3) | 78.4 (±0.3) | 99.9 (±0.6) | <.001 |
| Physical activity (METs-h) | 41.9 (±2.9) | 47.3 (±3.0) | 39.1 (±2.1) | 0.30 |
| Total energy intake (kcal) | 1890 (±30) | 2010 (±30) | 2000 (±30) | 0.03 |
| Dietary potassium (mg) | 2740 (±50) | 2790 (±50) | 2750 (±40) | 0.93 |
| Dietary calcium (mg) | 890 (±20) | 920 (±20) | 920 (±20) | 0.28 |
| Dietary magnesium (mg) | 299 (±6.6) | 299 (±6.0) | 290 (±4.3) | 0.20 |
| Dietary sodium (mg) | 3100 (±80) | 3280 (±60) | 3440 (±60) | <.001 |
| Dietary vitamin D (mcg) | 4.8 (±0.3) | 5.2 (±0.2) | 4.6 (±0.2) | 0.49 |
| Dietary total fat (g) | 70.8 (±1.6) | 77.8 (±1.3) | 80.0 (±1.7) | <.001 |
| Dietary saturated fat (g) | 22.8 (±0.6) | 25.3 (±0.5) | 26.1 (±0.6) | <.001 |
| Dietary cholesterol (mg) | 240 (±10) | 281 (±8.4) | 292 (±6.7) | <.001 |
| Race/Ethnicity (%) |  |  |  | <.001 |
| Non-Hispanic White | 80.9 | 80.4 | 76.6 |  |
| Non-Hispanic Black | 6.3 | 6.9 | 11.7 |  |
| Mexican American | 2.7 | 5.6 | 5.9 |  |
| Other ethnicity | 10.1 | 7.0 | 5.8 |  |
| Alcohol consumption (%) |  |  |  | <.001 |
| Never | 32.5 | 33.4 | 39.1 |  |
| < once a week | 28.1 | 27.4 | 35.9 |  |
| 1-2 days/week | 13.2 | 15.1 | 12.9 |  |
| 3-4 days/week | 9.4 | 9.4 | 4.9 |  |
| ≥5 days/week | 16.8 | 14.7 | 7.1 |  |
| Smoking status (%) |  |  |  | <.001 |
| Never | 46.8 | 49.5 | 50.3 |  |
| Former | 31.5 | 38.0 | 38.1 |  |
| Current | 21.7 | 12.5 | 11.7 |  |
| Season of examination (%) |  |  |  | 0.16 |
| November-April | 37.2 | 32.2 | 35.4 |  |
| May-October | 62.8 | 67.8 | 64.6 |  |
| Education (%) |  |  |  | 0.03 |
| <9^th^ grade | 7.1 | 8.0 | 8.6 |  |
| ≤High school graduate | 33.1 | 38.9 | 39.7 |  |
| >High school graduate | 59.8 | 53.1 | 51.7 |  |
| Hypertension (%) | 45.8 | 52.5 | 69.0 | <.001 |
| Diabetes (%) | 8.3 | 10.5 | 26.6 | <.001 |

^§^Geometric means (95% confidence interval) are presented.

Survey t-test for continuous variables and survey (Rao-Scott) χ^2^ test for categorical variables were used.

Supplemental table 2. Adjusted multiple logistic regression and 95% CIs of high pulse pressure by vitamin D concentrations in subjects classified by three BMI classifications

| BMI classifications | High PP |
| --- | --- |
| Total 25(OH)D | Fully adjusted model |
| Normal weight (< 25 kg/m^2^) |  |
| < 50 (nmol/L) | 1 (Reference) |
| 50~74.9 (nmol/L) | 0.57 (0.30, 1.06)^†^ |
| ≥ 75 (nmol/L) | 0.38 (0.21, 0.67)* |
| *p*-trend | 0.001 |
| Overweight (25~29.99 kg/m^2^) |  |
| < 50 (nmol/L) | 1 (Reference) |
| 50~74.9 (nmol/L) | 1.08 (0.72, 1.64) |
| ≥ 75 (nmol/L) | 1.02 (0.70, 1.50) |
| *p*-trend | 0.95 |
| Obese (≥ 30 kg/m^2^) |  |
| < 50 (nmol/L) | 1 (Reference) |
| 50~74.9 (nmol/L) | 0.91 (0.68, 1.24) |
| ≥ 75 (nmol/L) | 0.79 (0.56, 1.12) |
| *p*-trend | 0.18 |

**P*<0.05, ^†^*P*<0.1 compared with vitamin D levels < 50 nmol/L.

Fully adjusted model adjusted for age, sex, and race/ethnicity, education, season of examination, physical activity, alcohol consumption, smoking status, dietary covariates (intakes of total energy, potassium, calcium, magnesium, and sodium), height, weight, and diabetes.

Supplemental table 3. Adjusted multiple logistic regression and 95% CIs of high pulse pressure by vitamin D concentrations according to race/ethnicity groups

| Race/Ethnicity | High PP |
| --- | --- |
| Total 25(OH)D | Fully adjusted model |
| Non-Hispanic White |  |
| < 50 (nmol/L) | 1 (Reference) |
| 50~74.9 (nmol/L) | 0.92 (0.69, 1.21) |
| ≥ 75 (nmol/L) | 0.73 (0.55, 0.97)* |
| *p*-trend | 0.01 |
| Non-Hispanic Black |  |
| < 50 (nmol/L) | 1 (Reference) |
| 50~74.9 (nmol/L) | 0.60 (0.44, 0.82)* |
| ≥ 75 (nmol/L) | 1.10 (0.72, 1.70) |
| *p*-trend | 0.43 |
| Mexican American |  |
| < 50 (nmol/L) | 1 (Reference) |
| 50~74.9 (nmol/L) | 0.76 (0.55, 1.06) |
| ≥ 75 (nmol/L) | 0.98 (0.48, 2.00) |
| *p*-trend | 0.74 |
| Other ethnicity |  |
| < 50 (nmol/L) | 1 (Reference) |
| 50~74.9 (nmol/L) | 1.56 (0.80, 3.05) |
| ≥ 75 (nmol/L) | 1.91 (0.85, 4.31) |
| *p*-trend | 0.10 |

**P*<0.05 compared with vitamin D levels < 50 nmol/L.

Fully adjusted model adjusted for age, sex, and race/ethnicity, education, season of examination, physical activity, alcohol consumption, smoking status, dietary covariates (intakes of total energy, potassium, calcium, magnesium, and sodium), height, weight, and diabetes.

Supplemental table 4. Adjusted multiple logistic regression and 95% CIs of high pulse pressure by vitamin D concentrations according to season of examination

| Season of examination | High PP |
| --- | --- |
| Total 25(OH)D | Fully adjusted model |
| November-April |  |
| < 50 (nmol/L) | 1 (Reference) |
| 50~74.9 (nmol/L) | 1.14 (0.86, 1.51) |
| ≥ 75 (nmol/L) | 0.79 (0.49, 1.26) |
| *p*-trend | 0.28 |
| May-October |  |
| < 50 (nmol/L) | 1 (Reference) |
| 50~74.9 (nmol/L) | 0.76 (0.58, 1.00)^†^ |
| ≥ 75 (nmol/L) | 0.71 (0.55, 0.93)* |
| *p*-trend | 0.02 |

**P*<0.05, ^†^*P*<0.1 compared with vitamin D levels < 50 nmol/L.

Fully adjusted model adjusted for age, sex, and race/ethnicity, education, season of examination, physical activity, alcohol consumption, smoking status, dietary covariates (intakes of total energy, potassium, calcium, magnesium, and sodium), height, weight, and diabetes.
